# Perceptions of vision care following neurological impairment: a qualitative study

**DOI:** 10.1186/s12913-024-11079-9

**Published:** 2024-05-14

**Authors:** Kerry Hanna, Elizabeth Lomas, Stephen Rimmer, Fiona Rowe

**Affiliations:** 1 School of Health Sciences, University of Liverpool, Liverpool, L69 3GB UK; 2 Patient and Public Involvement Representative, Liverpool, UK; 3 Institute of Population Health, University of Liverpool, Liverpool, UK

**Keywords:** Visual impairment, Loss, Neurological impairment, Qualitative, Vision care, Inequalities

## Abstract

**Background:**

Visual impairment is a common consequence of neurological impairments, and can impact a person’s ability to undertake everyday tasks, affecting their confidence and mental health. Previous qualitative research in the UK has shown inequalities to exist where patients are accessing vision care after stroke, but little is known around the experiences of accessing vision care following other neurological impairments, and a lack of national guidelines prevent standardised care planning. The aim of this qualitative study is to explore the perceptions of vision care after neurological impairment, and to identify possible inequalities and support mechanisms, where it has been possible to access vision care.

**Methods:**

University ethical approval was obtained, and adults with a visual impairment as a result of a neurological impairment were offered an in-depth interview to explore their vision care experiences. Data were collected between April and November 2021 and analysed using iterative, thematic analysis (TA), informed by a social constructionist ideology.

**Results:**

Seventeen participants were recruited. Three overarching themes were conceptualised in relation to the participants’ perception of vision care: Making sense of the visual impairment; The responsibility of vision care; and Influential factors in care quality perception.

**Conclusion:**

Inequalities were noted by participants, with most reporting a lack of suitable vision care offered as part of their neurological rehabilitation. Participants were thus burdened with the task of seeking their own support online, and encountered inaccurate and worrying information in the process. Participants noted changes in their identity, and the identity of their family carers, as they adjusted to their vision loss. The findings from this research highlight a need for clinicians to consider the long-term impact of vision loss after neurological impairment, and ensure patients are provided with adequate support and information, and appropriate referral pathways, alleviating this patient burden.

**Supplementary Information:**

The online version contains supplementary material available at 10.1186/s12913-024-11079-9.

## Background

Visual impairment is a common consequence of neurological impairments, such as neurodegenerative conditions and acquired brain injury (ABI), including traumatic and non-traumatic brain injury, such as stroke, brain tumours or infection [1]. The prevalence of visual impairment following all types of ABI varies significantly in the literature, as it depends on the type, location and severity of the injury, however researchers have estimated that the prevalence of subjective visual impairment can exceed 70% of patients [2, 3]. This figure may be underestimated, however, as some patients do not describe their problems as “vision related” [4]. The prevalence of vision impairment in neurodegenerative conditions such as multiple sclerosis, Alzheimer’s disease, and Parkinson’s disease, has been reported between 1.7–58% [5–7]. However, studies often consist of small numbers, with some authors considering visual acuity decline as the only measure of vision impairment [3, 5], without considering other visual impairments that may occur following neurological impairment such as, difficulties with visual field loss, ocular motility disorders, and visual perceptual deficits [8, 9].

The impact of living with visual loss following neurological impairment can be far reaching. Recent research reported complications with visually impaired (VI) persons returning to work or driving, undertaking everyday tasks, and socialising, with subsequent effects on confidence and mental health [10, 11]. Moreover, neurological impairments often result in additional comorbidities or health complications that the person must learn to overcome, such as speech, swallow and gait issues, alongside their new visual impairments, impacting on their engagement with rehabilitation and quality of life [12, 13].

Due to the wide-reaching visual loss experienced following neurological impairments, and the complexity of assessing visual impairment in addition to other generalised disabilities, orthoptists are best placed to diagnose and manage visual disorders [14]. However, previous qualitative research in the UK has shown inequalities to exist where patients are accessing vision care after stroke, with care reportedly inconsistent across hospitals, and the visual impairments impeding patients from attending clinic appointments [12]. In addition, there is research to suggest that orthoptists are not always included in the care planning for these patients [15]. National guidelines exist in the UK, stipulating recommendations for appropriate orthoptic visual care after stroke [16], however similar guidelines are lacking for other types of neurological impairment. Therefore, clinicians assessing and treating patients within these fields are unsupported in detecting and managing visual impairments, meaning patients’ visual impairments may be missed or misdiagnosed.

Whilst research has expanded in recent years to explore the inequalities facing VI patients in accessing visual care following stroke specifically, it is possible that inequalities exist within the care pathway of other neurological conditions but this is yet to be established in the published literature. By providing a better understanding of inequalities facing people in accessing vision care following a wider range of neurological impairments, vision services can aim to adapt and improve their delivery, ensuring maximum engagement from patients. Therefore, the aim of this qualitative study was to explore the perceptions of vision care after neurological impairment, and to identify possible inequalities and support mechanisms experienced by people, where it had been possible to access vision care.

## Methods

### Aim, design and setting of the study

Qualitative data were collected between April and November 2021, which aimed to explore the perceptions of vision care after neurological impairment. Original research funding was granted in 2020, however due to the pandemic restrictions at that time, all non-COVID-19 related research projects were temporarily halted at the University. The project was restarted in 2021 following an amendment to the ethics application to allow for remote individual, semi-structured interviews. A topic guide was used to support the interview discussion (see Appendix 1). Participants were initially asked demographic background questions, and were then asked to discuss their perception of visual care following their neurological diagnosis/impairment, and throughout subsequent treatment. In addition, participants were asked to discuss the impact that their neurological condition, and subsequent care received, had on their wellbeing.

Participants were offered an interview via an online video call platform, or a recorded telephone call, dependent on the participant’s preference. Interviews were conducted by one researcher (KH), a female academic researcher and qualified clinical orthoptist. No one else was present for the interview, other than the interviewees and the interviewer. One outpatient department in an acute neurological centre in England, and various national charity organisations across the UK, advertised the study for recruitment. The study information was shared with organisations, and further information was further posted on social media. Interested participants then contacted the principal investigator via email and an approved participant information sheet and consent form were emailed to the participants, and re-read again prior to taking consent. Formal, written consent was obtained prior to an interview date being arranged. Further informal, verbal consent was taken before the interview commenced, which was also audio-recorded, as per the approved ethical protocol. In addition, the interviewer made confidential field notes at the time of interview, to ensure each topic was discussed thoroughly. Interviews lasted on average 34.75(± 12.1) minutes.

The COREQ checklist was used to guide the reporting of this research [17], See Supplementary Material 1.

### Participants

Eligibility for the study included adults with a vision impairment as a result of a neurological impairment, such as a traumatic brain injury, a stroke or a degenerative neurological disease. Visual impairments could include acuity loss, ocular motor impairments and/or double vision, visual perceptual disorders, visual field loss, or a combination of impairments. Participants had to be aged ≥ 18 years and residing in the United Kingdom (UK). Due to the potential severity of participants’ visual impairment, a second person (such as a spouse, partner or sibling) was permitted to take part in the interview as well, if it was deemed that the participant would not be able to complete the interview otherwise.

### Data analysis and epistemology

Iterative, thematic analysis (TA) was employed, informed by a social constructionist ideology, exploring the lived experiences of the participants through language and narrative. Thus, open questioning was used to generate responses that were actively constructed by the individuals through personal accounts [18]. TA was chosen for this research as it offers a flexible and in-depth way of exploring the research question, and allows for consideration not only of the perspectives of the individuals, but the meanings behind their choices and any potential impact on these meanings from wider society [19]. A reflexive TA strategy was followed, based upon the six-step model outlined by Clarke and Braun [20]. The researchers acknowledge that reflexive thematic analysis does not support the concept of data saturation as a guiding principle for qualitative sample size [21]. Thus, interviews were immediately transcribed and coded, so that meaning (instead of prevalence) of codes could be carefully considered. Recruitment ceased when it was determined by the research team that no new meaning was derived from the interviews.

Three interviews were transcribed by KH, to allow for immersion within the data and to better support later conceptualisation of themes [22]. The remaining interviews were transcribed by an experienced typist at the University, and anonymised by one researcher (KH) whilst checking for accuracy. Transcripts were not returned to participants for accuracy as no recording errors were identified. Transcripts were then blindly coded by two researchers (KH and EL), who are experienced qualitative researchers. In addition, two transcripts were double coded, to provide a 10% quality check.

### Reflexivity

Reflexivity enhances the quality of research, by disclosing the researchers’ position(s) and considering similarities or differences with the recruited participants. All authors are female, clinical orthoptists with professional academic backgrounds (academic teaching and research). FR is a Professor of Orthoptics with extensive experience in the field, who mentored the lead researcher on this project. KH is a postdoctoral researcher with a research interest in addressing health inequalities, and conducted the interviews. KH and EL analysed the data and are experienced qualitative researchers. All authors have contributed their perspectives to this research through agreement of overall findings and approval of the manuscript.

One public advisor with a visual impairment following acquired neurological impairment, contributed to the study development by overseeing the study protocol, qualitative methodological plan, and preliminary analysis. This insight helped to inform the research and ensure the findings were grounded within the reality of living with visual impairment following neurological impairment.

## Results

### Demographics

Seventeen participants (15 VI participants with an acquired neurological impairment, and one dyad consisting of a VI individual with their partner) completed an interview. Table 1 shows the demographics of included participants.

**Table 1:** Demographics of included participants

Demographical information	Participants (*n* = 16 + 1 partner)
**Gender**	
Female	11 (64.7%)
Male	6 (35.3%)
**Ethnicity**	
White British	15 (88.2%)
White Other	1 (5.9%)
Black Asian and Minority Ethnic	1 (5.9%)
**Relationship with participant** (if interviewed in a dyad)	
Partner	1 (100%)
**Visual impairment(s)**:	
Visual field loss	10 (62.5%)
Visual perceptual disorders	5 (31.3%)
Visual acuity loss	4 (25.0%)
Ocular motility impairment	4 (25.0%)
Other^1^	2 (12.5%)
**Acquired neurological impairment**:	
Stroke (primary)	7 (43.8%)
Stroke (secondary)^2^	2 (12.5%)
Multiple sclerosis	2 (12.5%)
Traumatic brain injury	2 (12.5%)
Basilar artery migraine	1 (6.3%)
Meningioma	1 (6.3%)
**IMD Decile** ^3^	
1 (most deprived)	0
2	3 (17.3%)
3	2 (11.8%)
4	2 (11.8%)
5	4 (23.6%)
6	1 (5.9%)
7	3 (17.3%)
8	0
9	1 (5.9%)
10 (least deprived)	1 (5.9%)
	**Mean (SD), [Range]**
Age (years)	44.1 (11.2), [29–74]
Years since neurological impairment diagnosis/onset	5.0 (5.4), [0.9–21]
Years since visual impairment onset	5.1 (5.5), [0.9–21.5]
Years of education	16.9 (2.7), [12–22]

Table legend: Many participants reported multiple visual impairments, therefore more visual impairments have been reported above than number of VI participants recruited to the study. ^1^Other visual impairments included: Migraine with brainstem aura, eye fatigue and eye strain. ^2^Stroke secondary to heart surgery, and Moya Moya disease. ^3^Index of Multiple deprivation score [23]

### Thematic analysis

Following analysis of the transcripts, three central themes were conceptualised from the data: (1) Making sense of the visual impairment; (2) The responsibility of vision care; and (3) Influential factors in care quality perception. Table 2 shows the themes and subthemes following analysis.

**Table 2:** Themes and subthemes following thematic analysis

Theme	Subtheme
1. Making sense of the visual impairment	• The emotional impact of the diagnosis • Fear, uncertainty and adjusting to the diagnosis
2. The responsibility of vision care	• The patient burden in seeking support • Research, social media, and self-seeking help • Changes to self-identity
3. Influential factors in care quality perception	• The staff-patient relationship, and power balance • Communication, and good care versus good luck • Receiving vision care during the COVID-19 pandemic

### Making sense of the visual impairment

#### The emotional impact of the diagnosis

Participants spoke of the initial shock when diagnosed with a neurological impairment, and subsequent visual loss. The clinical diagnosis was unexpected and as such, the participants had very little prior awareness of the condition(s) or long-term outcomes. The lack of previous knowledge resulted in confusion and worry, as they struggled to come to terms with the loss and change in lifestyle.

*…with having no understanding of traumatic brain injury and they [health professionals] never said anything…they didn’t seem to know I was in post-traumatic amnesia…it was [from] me personally googling…and coming to the realisation myself a year down the line that I suffered a very severe traumatic brain injury****ID04***

*I had a stroke…completely unexpected, there were no FAST symptoms whatsoever…I instantly lost part of my vision on the right-hand side…****ID05***.

*…you go through the various stages, it’s that shock, then the anger… Sight is a primaeval sense that people fear losing****ID07***.

Due to the limited awareness of visual loss and how this may present, the language participants used to describe their visual impairments was often unique to their own experiences and their own interpretation of their vision (as opposed to the terminology used in clinical and academic settings). Their visual description was often blended with other neurological deficits, such as dizziness, fatigue and speech problems, highlighting the multitude of issues presenting with neurological impairment. Overall, participant accounts expressed a sense of fear and concern where health professionals were not offering a formal visual diagnosis initially, despite participants describing visual loss in their own words.

*my third nerve would buzz and then I could definitely link it to, if it buzzed then the next day I could see more, I could move my eye more…****ID04***

*… it was just like stars in front of my face, it felt like when I was about to faint and I’d been saying for 2 weeks that I was having some problems with my sight and nobody had done anything…it was then found out that I had the visual field loss****ID06***

*[I get]…occasional clouded vision if I get really, really tired. It’s the focussing thing generally because one eye moves faster than the other…now and again [I get]…double vision with my forward [vision]…****ID02***

*[I get] pain…dizziness, forgetfulness, broken words and stammering. So, I’ll forget to speak…and I can walk into things and I can become very forgetful whilst I’m talking to somebody…****ID01***

### Fear, uncertainty and adjusting to the diagnosis

Participants reported confusion around their diagnosis, often due to a lack of clear information and a collection of misdiagnoses from a range of neurological conditions until health professionals were certain of a true cause. This, at times, led to a lack of trust around the final diagnosis, which in turn led to struggles in adjusting.

*At first, they [health professionals] didn’t know what the condition was, they assumed it was maybe a stroke, but then went to Meniere’s disease…and, a professional…did tests on me and said it’s basilar artery syndrome…It [the condition]’s not stopped [and] normally it stops and gives people some respite****ID01***

*it really upset me, it made me feel like why are they not listening to me…I knew that something was wrong with me…that distressed me more, the fact that I knew something was wrong…****ID06***

Participants were unclear of the long-term outcome of their visual loss, and whether they would recover and be able to return to activities such as driving. Evidence of adaptation was apparent in some areas of life, but not in others. In most cases, participants were using learned methods to get by, but were still aware of challenges in their day-to-day life due to their ongoing visual impairments.

*I wouldn’t be allowed to drive unless I wore an eye patch…that would be a big thing…I don’t like the idea of driving with an eye patch over the right eye… because I was hit on the right side [road traffic accident] I don’t like the idea of not being able to see on the right-hand side.****ID04***

*…things like exercise…now I have to take a step back and just do 10 rather than 20 min…I did have a…panic attack…I felt overexerted and…I knew in the back of mind I’d already had a stroke, I just didn’t want to risk it****ID10***

*I just make sure I cross the road properly… I don’t wear headphones outside now…it’s getting a little better but I am being more cautious like, making sure that I have someone on my left if I’m outside…****ID13***

In addition, navigating life after visual loss resulted in fear for some, as they worried about how others would perceive them. Participants described new, self-enforced, social limitations as a form of adapting, due to feelings that others would misinterpret the physical consequences of their neurological and visual impairments.

*I can I look like I’m drunk even though I don’t drink…. So, I’m a bit self-conscious of that*.

*so I don’t like to go out…I’ll tend to be quiet and not speak or if I do speak it’s quite short which comes across rude and it’s not me being rude it’s just being over cautious and not wanting to look like a bit of an idiot.****ID01***

*I hate going out…I don’t like going for shopping…because I bump with people because…I cannot judge distance as well…sometimes I fall down because I didn’t see the pavement, which makes me very anxious…when I bump with people [they] think oh I just didn’t see [them] but it’s a problem with [my] vision.****ID03***

### The responsibility of vision care

#### The patient burden in seeking support

Participants reported a range of people responsible for supporting them in receiving their visual care; however, primarily this task resided with the participant themselves. The participants reported cases where the responsibility to seek out advice and support resulted in them asking for help, and accepted blame for lack of care received where they failed to mention their symptoms at appointments.

*I didn’t know it [neurological impairment] was going to affect my [vision]…I didn’t realise it would visually impair me. So, I didn’t raise it with my consultant… they’ve not provided me any support for my eyesight loss but only because I’ve not told them.****ID01***

*they [hospital staff] literally said bye we will see you later, they gave me no information at all, no suggestions of phone numbers for [charity organisation] or anything at all on things I could do to try and improve****ID06***

Family support was a frequent point of discussion, with participant accounts illustrating the importance of advocacy, and the inequity that would arise from not having family or friends to advocate on their behalf, particularly in the earlier stages of diagnosis. Participants acknowledged that they would not have received the level of visual support and information that they managed to attain without the persistent support of a family member acting on their behalf. Again, these accounts portrayed a gap in the care system, whereby adequate information was not provided by a formal healthcare professional at the time of neurological/visual diagnosis.

*…mum and dad were very supportive but obviously they don’t have any knowledge of any of the conditions…they didn’t think I’d have any problems because of my head injury…they weren’t told anything [from the health professionals]****ID04***

*I get on with it but I would feel terribly sorry for somebody who didn’t have…a very supportive family, very supportive friends…****ID07***

Peer support groups were discussed as a means of gaining more information around the newly acquired diagnoses, and learning adaptation strategies from others with the same experiences. However, participants were again left with the responsibility to find suitable groups themselves, and many reported a lack of suitable services available. Some reported feeling they were not appropriate members for such groups, as they did not perceive their visual impairments to be as severe as others in the group. Similarly, where it was felt that the group discussions were frequently negative and unhelpful, the participants disengaged.

*it’s always really useful when you meet other people who have either the same or a similar condition…you can say to somebody…things that you wouldn’t really bother asking a doctor. I went along to some local groups, which I didn’t stick with because I found them a little bit depressing. I’m quite a positive person and…obviously because the diagnosis, it wasn’t what I thought was a very positive atmosphere****ID02***

*I’d researched stuff off my own back, no medical professionals gave me anything. I joined the [charity organisation] Facebook groups and…it was only in there that I started getting suggestions from other people in the same situation…if I’m honest I felt like a fraud because I was partially sighted whereas everyone in my group was totally blind…I remember being like this is this not going to help me at all****ID06***

#### Research, social media, and self-seeking help

With frequent misdiagnoses, misinformation or a lack of formal visual care, many participants turned to internet searches to explain their symptoms, and to seek evidence of long-term outlook. However, participants readily acknowledged the limitations of self-seeking information online, due to the vast quantity of unevidenced, misinformation available. Online charity organisations can provide support and advice, if participants are able to find these websites. In all cases, participants reported a desire for clear and formal information to be signposted by the healthcare professionals, to limit the burden placed upon them and the possibility of accessing misinformation.

*…I do read a lot about [neurological condition], in the beginning…I just wanted to know more about it…I’m very aware that there’s a lot of information out there that isn’t maybe quite accurate so I found the [charity organisation] very useful because I’d see that as quite a trusted source****ID02***

*…I did loads of research…it was just very difficult because I’m not a doctor so…sometimes…you go down that rabbit hole [and] it makes you feel worse…I’d rather hear it from someone [who] at least has a bit of training on it rather than just some guy on YouTube****ID10***

In addition, where visual care was lacking, participants reported using the internet to seek out research studies or non-national health service (NHS) centres conducting work into visual loss due to their neurological impairment. Enrolling in research projects allowed participants to find out more information concerning their eyesight, and to hopefully manage the condition.

*I’m doing this research, it’s free, they’ll even pay you… So, its free rehab they’re paying you to do…and people [from peer support group] were still like oh no it’s a little bit too far for me, and I’m thinking God I would have travelled to America to get my eyesight back****ID10***

*No [I didn’t receive visual treatment] from the hospital but I did another research thing, a student from the University… there was a computer-based thing where you had to hit keys to move things about…I still keep that up because…I think it helps but I’m not sure…****ID02***

However, in some cases, participants reported high personal costs incurred from embarking on treatments found online that are not available through the NHS, and were later found to be unsuitable. This highlights a significant inequality in care, where patients spend unnecessary money trying to seek care, which could be avoided through clear advice from health professionals around their visual prognosis. Participant accounts described a sense of desperation and urgency in trying to recover their lost vision through any means possible, and the understandable disappointment of paying for treatments that had little or no effect on their vision.

*I found out about [private vision clinic]…I thought I might as well give it a go because I’ve got nothing to lose, other than money…I think it said I’d improved like 5% but I think…that 5% is for me knowing the task more, I don’t think it’s from my eyesight improving****ID06***

*looking back now I think it was a bit of a shamble, it’s this doctor on YouTube and she claims that she can get up to 15 to 80% of your vision back if you’ve had a stroke and…It was almost 10 thousand dollars, so that’s how much I wanted my eyesight back****ID10***

#### Changes to self-identity

For many, interview discussion naturally incorporated comparison of their lives pre and post-visual impairment. Changes to their lives often included loss of employment, limitations in driving and using transport, and relationship breakdowns in their personal lives. This discussion appeared to emphasise the scale of loss and significant changes to their normal day-to-day living.

*I was a very healthy person. I cycled over eight miles a day, I drank [alcohol] little, I didn’t smoke and nor did drugs [pause]. I lost my job from [neurological impairment]…most of my friendship group didn’t know how to deal with it…I now say I’ve got no friends because they don’t come round anymore…****ID01***

*I had to apply reapply for my [driving] license and I told [the driving agency] I’d had a stroke…and because my vision…it was revoked…I completely lost my independence through all of that****ID05***

*I was very independent…I had to move back in with my parents for them to look after me, and having to ask them to drive me around everywhere, it took me back to being 15 again…I just I stopped socialising****ID06***

In light of these changes, many of the participants reported a new identity that formed from their visual loss. Many enrolled in a number of research projects to better understand their vision loss, and viewed this as a new role; supporting the growing evidence base and preventing similar issues affecting people with their condition. Furthermore, a change in identity was noted in the family members who became active carers for the participants. They took on the role of procuring food, medication and actively searching for relevant support groups online. It was reported that family carers took time to accept the label of their new role as carer for their loved one.

*I want to be able to get back to normal life…but I spend my day googling exercises I can try and finding people that have gone through similar things…But there is hope that it might improve.****ID14***

*my wife has only in the last six months decided or accepted that she is in fact my carer because she gets my medication, she drives me around and provides food, but she’s only just accepted that****ID05***

*I think that anyone that hasn’t got a partner as committed and interested as [partner], they’d struggle a lot. I don’t know where I’d be now, if it was not [for partner] to help [with sorting] my food out, and like make those little changes…****ID16***

### Influential factors in care quality perception

#### The staff-patient relationship, and power balance

Where visual care was offered to participants following their neurological diagnosis, an apparent power imbalance between the clinicians and the patient was described. Often this appeared to stem from the participant’s lack of prior knowledge around the neurological and/or visual impairment, which left them reliant on the information offered by the clinicians.

*when the appointments were happening around my vision I would say I was too tired and didn’t have the mental capacity, so I wasn’t looking at anything to do with the vision, I was just leaving it to what the hospital told me.****ID04***

*they could have given me leaflets…I would have liked that information…even when they [health professionals] were talking to me I tried to write notes in the phone and they told me off and they were like oh don’t worry we will send it in a letter but the discharge thing was all in their language…****ID13***

Due to the persistent frustration of living with impairments and not receiving the appropriate care, some participants spoke of instances where they reclaimed power in the staff-patient relationship. Unfortunately, this often resulted from formal complaints to the hospital in the hope that a referral to appropriate vision services would be made, with participants describing a breaking point in their mental wellbeing.

*I rang up [patient advice and liaison service] at the hospital…I hadn’t heard anything for 15 or 16 months, and all of a sudden, I had a phone call… and then they referred me to the orthoptist…****ID06***

*it took a lot for me…I was getting really frustrated…I was ringing around and [saying] listen, you know there’s something wrong with my eyes can somebody refer me…I remember being really annoyed…[it was] after that when I had to go and see the specialist****ID10***

#### Communication, and good care versus good luck

Some participants praised the vision care offered to them. The factors that influenced a perception of good care included visual care being offered early after diagnosis, and consistently thereafter. Further factors of good care included considerations for wider support, including that for the family carers, and being provided with sufficient time to ask questions, and have these adequately addressed by the clinicians.

*[I was offered vision care] quite early into the process…and that’s continued all the way through, every time I have an appointment at the [neuro] centre I see the vision lady as well [pause]. As a patient it’s that element of control…a lot of your life you don’t have control of anymore but you feel like you’re in control of your appointment, when you go and what you want, it’s very person centred.****ID02***

*The nicest person I had in the medical service…cared more for [my wife] as well [as me]. She was asking about how she [wife] was doing and she was looking for services for us…she realised that care wasn’t just for that person the care was for in the family as well****ID01***

A notable finding from this research is that when vision care was offered, even if it was not found to be particularly helpful in addressing their concerns, the participants frequently described themselves as “lucky”. Participants credited exceptional reasons for why their care was lucky, including clinicians being helpful in response to others failing to listen to them, or knowing hospital workers who could help chase appointments.

*…my eyes went funny again so I went to the hospital and [the consultant] was really not very nice with me at all…insinuating that I was a hypochondriac…but my GP has been absolutely fantastic [helping with vision care referral]…I count myself lucky that I’m with them****ID09***

*…it was a struggle just to get the appointments…I remember getting so frustrated because I was ringing and ringing and ringing and I eventually went in [to hospital] and thank God I actually knew one of the nurses…I said listen can you have a look at my file and just find out why am I waiting so long for an appointment…****ID10***.

*I think there needs to be more like connection with services…I’m lucky because I work in a hospital so I’ve referred myself [to the clinic]…so, I’m lucky****ID13***

These participants further offered suggestions for how they would have liked to receive their visual care. Suggestions included timely, targeted advice soon after their visual diagnosis, and improved knowledge of visual consequences by health professionals. In instances where the neurological impairment impeded participants from taking in information provided by health professionals, a preference for (lay) information to be shared with family was suggested.

*I would say there appears to be, the fact that the paramedics didn’t realise you can have a stroke without FAST symptoms…particularly for hemianopia and none of them ever pick up on it…I would have thought if you’re going into any kind of medical training you would have [that] knowledge****ID05***

*once you’ve been diagnosed there should be somebody really outside the door with a leaflet. I do think it should be factored in. Even if it’s patient services liaison just sat outside the hallway just to say…what your condition is…****ID01***

#### Receiving vision support during and since the COVID-19 pandemic

For most participants in this study, their neurological and visual care had been ongoing for many years. Therefore, conversation naturally arose regarding the care received during the time of the COVID-19 pandemic, as participants were able to make contrasting observations from the time before and after pandemic restrictions. Additional inequalities in accessing care during pandemic restrictions were reported, including delays in receiving care, the transition to telemedicine, and an inability to see primary care physicians as a first point of contact when symptoms have changed.

*I’ve got nobody to help…[I’m] just not going to know what’s out there and what help I can get. I would say…because of COVID my aftercare was absolutely lacking for everything****ID11***.

*I’ve got another telephone appointment at the end of this month…I know COVID’s still going on but the levels are drastically dropping…I’m a slight worried that this may be the way most consultants now go…I just find telephones quite rude…if I’m having a conversation with somebody I can see how that person…reacting and talking…and [if] I’m unshaven and my clothes are hanging off and I’m smelling a bit they’ll say clearly I can see you’re not [doing well].****ID01***

Where participants noted that communication from health services during the pandemic was lacking, this furthered previous notions that the burden of care rested with the patient. The participants were left to chase hospital appointments and seek the help they needed when their vision was evidently deteriorating.

*I didn’t receive any letter from [the hospital] and I tried to chase them up. I tried to call them and no answer, and I sent them an email, no answer. And my vision gets worse…it’s not really good communication with patient, I know it’s COVID but this is about the health and about the vision, it’s about my life to be honest.****ID03***

*When lockdown happened…I rang up my doctors in tears and I was just like I can’t do this anymore, I’m having no help, I’m having no support. So, they then referred me to the local low vision people…all these were just purely from me ringing the doctors being like I can’t do this anymore****ID06***

Moreover, the impact that the pandemic restrictions had on daily life affected access to peer support groups and activities for VI people. Despite restrictions lifting, it was evident that some services did not reopen to the same level as before the pandemic, and for some, the confidence and enjoyment lost during this time could not be recovered. This resulted in further isolation and loss of social interaction.

*…before COVID [I went] to a walking group…and often we would need to get public transport… But once we got into COVID they started saying you can’t sit next to someone else and they would tape [seats] off. Even though that [restriction]’s been removed, I certainly [don’t] have the confidence to go on public transport of any description****ID05***

*…then COVID happened and I think it’s hampered my recoverybecause I just feel like I can’t get back to being the old me…I can’t take my daughter to nursery, I have to get…taxis to work and that’s like taking your life in your hands…it’s that horrendous, I just can’t look [due to vision impairment]…it feels like I’m a burden****ID11***

## **Discussion**

The findings from this qualitative exploration of participants with vision impairment as a result of neurological impairment, identified inequalities in the vision care pathway. These inequalities contributed to the participants’ inability to make sense of their visual impairment, the personal burden of responsibility in seeking vision care, and an imbalance in the staff-patient relationship due to poor communication and lack of adequate and equal care offered after neurological diagnosis.

Previous qualitative research with stroke survivors specifically reported similar inequalities in perceptions of the vision care pathway after diagnosis, including a lack of early visual assessment and subsequent management strategies [24]. This research was conducted 10 years prior to the current study, highlighting a lack of significant change around the quality of vision care offered to patients, and further highlights that lack of suitable vision care is not specific to stroke patients, but impacts other brain injury populations also.

Our study did not use discourse analysis when considering how participants described and understood their visual loss. However, the language used by participants when discussing visual impairments was noticeably unique in description, and did not easily reflect their clinical diagnoses. Previous research found patients to use a wide range of descriptors for symptoms of dry eye, glaucoma and macular degeneration [25–27], with the current findings adding that symptoms of neurological visual loss, such as double vision and visual field loss, are also described in a range of different ways by people with no previous awareness of such problems. Visual impairments following neurological impairment are often deemed “invisible” [28], with non-eye-trained clinicians relying mainly on patient reported symptoms of visual loss to make appropriate diagnoses and/or onward referrals [29]. Thus, differing descriptions of symptoms could impact patients in receiving a timely diagnosis and referral to vision services, as described in this research, in cases where clinicians cannot clearly identify the visual problems. Further evidence has shown that more detailed descriptions emerge where patients are previously informed of their condition from a trained eye professional [27]. Therefore, it is clear that patients require adequate information from trained professionals, such as orthoptists, in understanding their visual conditions. This will not only support clinicians in making appropriate diagnoses, but will support the patient in coming to terms with their visual loss, and identify appropriate online information or support groups targeted to their visual condition.

Another notable finding from this research was the frequent reference to vision care being “lucky”, despite otherwise negative descriptions of poor and delayed care. Previous ethnographic research reported descriptions of luck to emerge where patients received good news at an outpatient consultation [30]. However, in the case of our current study participants, visual impairments did not recover and treatment was rarely offered. Their perception of luck often related to a clinician listening to their needs, or making an appropriate referral (months or years after their initial onset). This perception of “lucky” care may reflect the staff-patient power imbalance identified from this study, whereby participants were dissatisfied with their care but did not always feel empowered to say so. This research further highlighted instances where participants eventually attempted to take back power after long-term frustration, through complaints made to health services. Similar instances of power imbalance have been noted previously with VI older adults, due to a lack of knowledge and self-reliance [31]. The authors suggested that professionals should be made aware of the range of unmet needs facing these patients, and empower them to disclose challenges during consultations [31]. Thus, it is first important to ensure patients are offered adequate and timely visual care, so that they can then be offered the opportunity to say when the care plan is not suitable for their needs, and avoid instances of high stress and hospital complaints in order to receive such care.

Changes in self-identity following neurological and visual impairments have been discussed previously in the literature [12], with affected people frequently exhibiting examples of “impression management” to protect their character [32]; creating a persona that reflects the important qualities of their life before their neurological diagnosis. The participants in this research frequently described changes to their lives after their neurological diagnosis that often centred around adapting (where possible) to their visual impairments, and becoming actively involved in research studies and peer support groups. This finding concurs with previous research into stroke survivors’ adaptation of visual field loss, which suggested that adaptation is an individual process and changes to meet the needs of the environment and task, with the authors promoting that early diagnosis and management should be offered to these patients to support them in adapting [33].

In addition, our findings shed light on the importance of patient advocates following neurological impairment, through which most vision support was sought and the interests of the participants promoted where participants were unable to do this for themselves, particularly in the early stages after diagnosis. As a result, it was reported that the family/informal carers also experienced changed identities, as they became actively involved in caring for their loved one, procuring basic necessities and searching for helpful online information. Previous literature reviews have reported changed roles of family carers in patients with chronic disease and neurological impairments, such as dementia and stroke [34–36]. These caregivers experience losses to their previous relationship and feelings of carer burden [35]. However, little is known around the impact of caring for someone with a co-existent visual impairment after neurological diagnosis, highlighting an area for further research to be conducted.

An important finding from this research was that vision care was unequal across the participant experiences, with most reporting no offer of vision support. Unequal vision service provision has been noted after a diagnosis of stroke in the UK [15], but this finding shows similar issues exist across other neurological impairment diagnoses. Where vision care is not received, the burden is placed on the patient to find support. Previous evidence has shown the value of peer support groups for VI people, which enhances their ability to cope with visual loss and improve levels of wellbeing [37]. However, our current participants reported stress caused from filtering through a myriad of online information, often inaccurate or worrying, and tried multiple support groups until they found one that was suitable to their needs. A recommendation from this research is that health professionals should signpost appropriate information and organisations to patients, preventing them from becoming over burdened with this task. Resources and factsheets are already in existence to support patients in this way, and clinicians should make good use of these without further effort required (see Supplementary Material 2). Moreover, our participants described a sense of desperation to restore lost vision where possible, resulting in them enrolling in projects or treatments that were later found to be unsuitable. Offering patients supportive information around the prognosis of their visual loss early after diagnosis, could further prevent them spending unnecessary money. Participants in this research described losing their jobs, and being unable to return to work resulting in a lower income, therefore, spending money on treatments unnecessarily will only widen such inequalities.

Although it was not the focus of the research, it was inevitable that discussion around the impact to health services caused by the COVID-19 pandemic emerged, as interviews were conducted within this time period. Participants reported issues with telemedicine, and challenges in speaking directly with a healthcare professional. This finding has been reported in other areas of health and social care research, as patients may experience digital exclusion due to poor internet connection, complex technologies and concerns around data protection and privacy [38]. However, there is a lack of robust evidence exploring the impact of telemedicine during the pandemic in vision services specifically, despite reports that tele-consultations for orthoptic services rose substantially during this time [39]. Survey responses collected by orthoptists during the pandemic raised concerns around ethical and confidentiality issues, in addition to technological issues experienced when delivering this service [39]. However, conflicting survey evidence indicates that telemedicine can be a suitable service for vision care; namely for reporting on the results of previous assessments and ongoing monitoring [40, 41]. The authors noted, however, that participants reported issues with the description of clinical findings via telemedicine [41], although no further explanation was offered for this finding due to the nature of the survey data collection method, which lacked in-depth exploration to better understand the patient experiences. Comparably, however, our participants also described challenges in understanding information provided by the healthcare providers, often as a result of their other neurological symptoms. Furthermore, participants reported an unmet need whereby telemedicine did not offer them the correct environment to disclose sensitive information and anxieties, whilst it was felt that clinicians would not be able to identify physical signs of lack of coping via the telephone. Therefore, it appears that telemedicine may have a place in ophthalmic practice, where patients have already been diagnosed with their visual condition and are receiving ongoing monitoring. However, face-to-face appointments may serve a better purpose in supporting those with visual loss following neurological impairment, particularly where their diagnosis is unclear and problems may arise later when adjusting to their vision loss.

### Limitations

There was a lack of Black, Asian and Minority Ethnic (BAME) participants within the study cohort, due to the convenient sampling method used in this explorative research. Previous evidence reported inequalities within this population specifically, when accessing vision care in the UK, such as health information not being available in different languages [42], and an underrepresentation of Asian patients registered partially sighted [43]. Moreover, differences in demographics (namely age) between white British and BAME VI stroke survivors have been reported [44]. Therefore, further inequalities may exist within the VI BAME cohort following other neurological diagnoses, that should be explored in future research using targeted sampling methods.

A further limitation of this work could result from the addition of one caring dyad. Combining dyadic and individual interviews is not ideal as it could be argued that different forms of data collection can yield differing result. However, this inclusion allowed participants to take part in research who would otherwise not have been able to. In this case, the VI participant was reliant on their partner to support their access to/engagement with the remote interview. This is particularly important in research into health inequalities, as it is possible that these participants experience barriers to inclusion in other aspects of their visual care, which should be recorded.

## Conclusion

This research is one of the first to explore experiences of vision care in participants with a broad range of neurological impairments. Inequalities were noted in the vision care pathway, which was often inadequate or absent altogether from participants’ neurological rehabilitation. Where vision care is lacking, participants are burdened with the task of seeking their own support online, and encountered inaccurate and worrying information in the process. There is a risk of patients paying high costs for treatments that are not suitable to their condition, due to a lack of formal vision support offered to them, and their desperation to seek treatment. The findings from this research highlight a need for clinicians to consider the long-term impact of vision loss after neurological impairment on the patient and their families, ensuring patients are provided with adequate support and information throughout the remainder of their neurological care. In addition, participants noted a preference for patient-centred, face-to-face consultations where possible, where they can openly discuss the impact of their vision loss with clinicians. Telemedicine was not deemed a suitable method for vision care delivery following neurological impairment, although use of this service appears to have increased since the COVID-19 pandemic. This finding can help inform the optimum method for vision service delivery for patients with neurological impairment.

Participants further noted changes in their identity after diagnosis, as they adjusted, where possible, to their visual loss. In addition, it was noted that family members had to adapt to the role of an informal carer during this time. Little is currently known around the role and impact of caring for a person with co-existing visual and neurological impairments, warranting further exploration.

### Electronic supplementary material

Below is the link to the electronic supplementary material.

Supplementary Material 1

Supplementary Material 2

Supplementary Material 3

## Data Availability

The data that support the findings of this study are available on request from the author [KH]. The data are not publicly available due to ethical restrictions.

## References

[1] Bruns J, Hauser WA (2003). The epidemiology of traumatic brain injury: a review. Epilepsia.

[2] Goodrich GL, Kirby J, Cockerham G, Ingalla SP, Lew HL. Visual function in patients of a polytrauma rehabilitation center: a descriptive study. J Rehabilitation Res Dev. 2007;44(7). 10.1682/jrrd.2007.01.0003 18075950

[3] Theis J. Visual disturbances in acquired brain injury. NeuroRehabilitation. 2022. 10.3233/NRE-228010 35311723

[4] Berthold-Lindstedt M, Ygge J, Borg K (2017). Visual dysfunction is underestimated in patients with acquired brain injury. J Rehabil Med.

[5] Jasse L, Vukusic S, Durand-Dubief F, Vartin C, Piras C, Bernard M et al. Persistent visual impairment in multiple sclerosis: prevalence, mechanisms and resulting disability. Multiple Sclerosis Journal. 2013;19(12):1618 – 26.10.1177/1352458513479840. 10.1177/1352458513479840 23462348

[6] Mendola JD, Cronin-Golomb A, Corkin S, Growdon JH (1995). Prevalence of visual deficits in Alzheimer’s disease. Optometry Vis Science: Official Publication Am Acad Optometry.

[7] Hamedani AG, Abraham DS, Maguire MG, Willis AW (2020). Visual impairment is more common in Parkinson’s disease and is a risk factor for poor health outcomes. Mov Disord.

[8] Rowe FJ, Hepworth LR, Howard C, Hanna KL, Cheyne CP, Currie J (2019). High incidence and prevalence of visual problems after acute stroke: an epidemiology study with implications for service delivery. PLoS ONE.

[9] Armstrong RA (2018). Visual problems associated with traumatic brain injury. Clin Experimental Optometry.

[10] Heinze N, Jones L, Makwana B (2023). A rapid review of evidence relating to service use, experiences, and support needs of adults from minority ethnic communities along the eyecare pathway in the United Kingdom. Front Public Health.

[11] Demmin DL, Silverstein SM. Visual Impairment and Mental Health: Unmet Needs and Treatment Options. Clin Ophthalmol. 2020;14:4229 – 51.10.2147/opth.S258783. 10.2147/OPTH.S258783 PMC7721280 33299297

[12] Hanna K, Mercer D, Rowe F (2020). A qualitative exploration of the sociology of poststroke visual impairments and the associated health inequalities. Brain Behav.

[13] Park WL, Mayer RS, Moghimi C, Park JM, Deremeik JT (2005). Rehabilitation of hospital inpatients with visual impairments and disabilities from systemic illness. Arch Phys Med Rehabil.

[14] Rowe F, Walker M, Rockliffe J, Pollock A, Noonan C, Howard C (2016). Delivery of high quality stroke and vision care: experiences of UK services. Disabil Rehabil.

[15] Hepworth L, Rowe F (2019). Ten years on - a Survey of Orthoptic Stroke Services in the UK and Ireland. Br Ir Orthopt J.

[16] Intercollegiate Stroke Working Party (2023). National Clinical Guideline for Stroke for the UK and Ireland.

[17] Tong A, Sainsbury P, Craig J. Consolidated criteria for reporting qualitative research (COREQ): a 32-item checklist for interviews and focus groups. International Journal for Quality in Health Care. 2007;19(6):349 – 57.10.1093/intqhc/mzm042. 10.1093/intqhc/mzm042 17872937

[18] Thorogood N, Green J. Qualitative methods for health research. Qualitative methods for health research. 2018:1-440.

[19] Braun V, Clarke V. Using thematic analysis in psychology. Qualitative Research in Psychology. 2006;3(2):77-101.10.1191/1478088706qp063oa.

[20] Clarke V, Braun V. Thematic analysis: a practical guide. Thematic Anal. 2021:1–100.

[21] Braun V, Clarke V (2021). To saturate or not to saturate? Questioning data saturation as a useful concept for thematic analysis and sample-size rationales. Qualitative Res Sport Exerc Health.

[22] McMullin C (2023). Transcription and qualitative methods: implications for third Sector Research. Voluntas.

[23] National Statistics (2019). English indices of Deprivation 2019.

[24] Rowe FJ (2017). Stroke survivors’ views and experiences on impact of visual impairment. Brain Behav.

[25] Taylor DJ, Edwards LA, Binns AM, Crabb DP (2018). Seeing it differently: self-reported description of vision loss in dry age-related macular degeneration. Ophthalmic Physiol Opt.

[26] Nichols KK (2006). Patient-reported symptoms in Dry Dye Disease. Ocul Surf.

[27] Cross V, Shah P, Bativala R, Spurgeon P (2005). ReGAE 3: Glaucoma awareness and perceptions of risk among african-caribbeans in Birmingham, UK. DiversityinHealthandSocialCare.

[28] Falkenberg HK, Mathisen TS, Ormstad H, Eilertsen G. Invisible visual impairments. A qualitative study of stroke survivors` experience of vision symptoms, health services and impact of visual impairments. BMC Health Services Research. 2020;20(1):302.10.1186/s12913-020-05176-8. 10.1186/s12913-020-05176-8 PMC7158142 32293430

[29] Rowe FJ. Accuracy of referrals for visual assessment in a stroke population. Eye. 2011;25(2):161 – 7.10.1038/eye.2010.173. 10.1038/eye.2010.173 PMC3169216 21127506

[30] Schoenau MN (2022). Dominant restitution narratives of ‘being lucky’: an ethnographic exploration of narratives about operable lung cancer. Eur J Cancer Care (Engl).

[31] van der Aa HPA, Hoeben M, Rainey L, van Rens GHMB, Vreeken HL, van Nispen RMA. Why visually impaired older adults often do not receive mental health services: the patient’s perspective. Quality of Life Research. 2015;24(4):969 – 78.10.1007/s11136-014-0835-0. 10.1007/s11136-014-0835-0 25398494

[32] Goffman E (1959). The presentation of self in everyday life.

[33] Howard C, Czanner G, Helliwell B, Rowe FJ (2022). Adaptation to post-stroke homonymous hemianopia–a prospective longitudinal cohort study to identify predictive factors of the adaptation process. Disabil Rehabil.

[34] Eifert EK, Adams R, Dudley W, Perko M. Family Caregiver Identity: A Literature Review. American Journal of Health Education. 2015;46(6):357 – 67.10.1080/19325037.2015.1099482.

[35] Greenwood N, Mackenzie A (2010). Informal caring for stroke survivors: Meta-ethnographic review of qualitative literature. Maturitas.

[36] Greenwood N, Mackenzie A, Cloud GC, Wilson N. Informal primary carers of stroke survivors living at home–challenges, satisfactions and coping: A systematic review of qualitative studies. Disability and Rehabilitation. 2009;31(5):337 – 51.10.1080/09638280802051721. 10.1080/09638280802051721 18608402

[37] Van Zandt PL, Van Zandt SL, Wang A (1994). The role of support groups in adjusting to visual impairment in Old Age. J Visual Impairment Blindness.

[38] Anthony Jnr B. Implications of telehealth and digital care solutions during COVID-19 pandemic: a qualitative literature review. Informatics for Health and Social Care. 2021;46(1):68-83.10.1080/17538157.2020.1839467. 10.1080/17538157.2020.1839467 33251894

[39] Rowe F, Hepworth L, Howard C, Lane S (2020). Orthoptic services in the UK and Ireland during the COVID-19 pandemic. Br Ir Orthoptic J.

[40] Woodward MA, Ple-Plakon P, Blachley T, Musch DC, Newman-Casey PA, De Lott LB et al. Eye care providers’ attitudes towards tele-ophthalmology. Telemed J E Health. 2015;21(4):271 – 3.10.1089/tmj.2014.0115. 10.1089/tmj.2014.0115 PMC4378352 25635290

[41] Smith J, Hirst N, Yagan A (2023). A Quality Assurance audit of an orthoptic-led virtual Neuro-Ophthalmology Clinic. Br Ir Orthopt J.

[42] Scase MO, Johnson MR, editors. Visual impairment in ethnic minorities in the UK. International Congress Series. Elsevier; 2005.

[43] Pardhan S, Mahomed I (2002). The clinical characteristics of Asian and caucasian patients on Bradford’s low Vision Register. Eye.

[44] Rowe FJ, Hepworth LR (2021). The impact of visual impairment in stroke (IVIS) study–evidence of reproducibility. Neuro-Ophthalmology.

