# Supplementary material for: Perceptions of vision care following neurological impairment: a qualitative study
Source: BMC Health Serv Res. 2024 May 14;24:626. doi: 10.1186/s12913-024-11079-9 (PMC11095032; doi:10.1186/s12913-024-11079-9)
Supplement: Supplementary file 1 — Supplementary Material 1 [file 12913_2024_11079_MOESM1_ESM.docx]

Appendix 1: Topic guide

**Introduction**

Purpose: *“The purpose of this interview is to discuss the vision support offered to you following diagnosis of a neurological condition”*

**Question plan and topics**

1. **Neurological impairment(s) experienced**

*Can you tell me about the neurological problem you experienced and the date in which this first began?*

PROMPT: when did you first receive a diagnosis of …?

PROMPT: what problems did you experience as a result of this diagnosis? (physically, psychologically etc.)

1. **Vision impairment(s) experienced**

*What changes did you notice to your vision in relation to the neurological condition?*

PROMPT: what impact did your visual problems have on your quality of life?

PROMPT: did these problems change over time?

1. **Vision support offered, and quality/quantity of the support (if applicable)**

*What vision support was offered to you immediately after your neuro diagnosis?*

PROMPT: this may include referral to hospital eye appointments, home based therapy or information provided to help you understand more about your vision etc.

PROMPT: did you find this helpful? Which aspects exactly were helpful to you?

PROMPT: how soon after your diagnosis did you receive this support/advice?

PROMPT: is there anything else that could have been done at that stage to better support you?

1. **Service providers**

*Do you know which healthcare provider/professional offered you your vision care?*

1. **Help seeking behaviours**

*Did you ever have to personally seek additional information about your vision?*

PROMPT: what did you do, and did you find it helpful?

1. **Follow up support**

*Have you received any long-term support after hospital discharge?*

PROMPT: can you describe the care received?

PROMPT: How has your vision been long term?

PROMPT: Do you feel people with new visual problems need long term care/advice to help cope with the changes?

**Conclusion**:

*And finally, is there anything about your vision care after neurological diagnosis that we haven’t already mentioned?*

PROMPT: anything that was particularly helpful, or anything that you feel you would have needed but did not receive?
