# Supplementary material for: Perceptions of vision care following neurological impairment: a qualitative study
Source: BMC Health Serv Res. 2024 May 14;24:626. doi: 10.1186/s12913-024-11079-9 (PMC11095032; doi:10.1186/s12913-024-11079-9)
Supplement: Supplementary file 3 — Supplementary Material 3 [file 12913_2024_11079_MOESM3_ESM.docx]

**Supplementary material 2.** Signposting resources for vision support following neurological impairment

1. Headway, the Brain Injury Association. Vision problems after brain injury factsheet. <https://www.headway.org.uk/about-brain-injury/individuals/brain-injury-and-me/visual-problems-a-closer-look/>
2. The British and Irish Orthoptic Society (BIOS), Stroke and Neuro Rehab advisory group
3. <https://www.orthoptics.org.uk/resources/clinical-advisory-group/stroke-and-neuro-rehabilitation/>
4. The British and Irish Orthoptic Society (BIOS), Neuro orthoptics advisory group
5. <https://www.orthoptics.org.uk/resources/clinical-advisory-group/neuro-orthoptics-and-ophthalmology/>
6. The University of Liverpool – VISION Research unit: [https://www.liverpool.ac.uk/population-health-sciences/departments/health-services- research/research/vision/professional-resources/](https://www.liverpool.ac.uk/population-health-sciences/departments/health-services-%20research/research/vision/professional-resources/)
7. The Royal National Institute for the Blind (RNIB). Stroke-related eye conditions <https://www.rnib.org.uk/your-eyes/eye-conditions-az/stroke-related-eye-conditions/>
8. The Royal National Institute for the Blind (RNIB). Navigating sight loss <https://www.rnib.org.uk/your-eyes/navigating-sight-loss/>
9. The stroke Association. Vision problems after stroke <https://www.stroke.org.uk/effects-of-stroke/physical-effects-stroke/vision-problems-after-stroke>
